# Purified *Monascus* Pigments: Biological Activities and Mechanisms of Action

**DOI:** 10.1021/acs.jnatprod.4c01008

**Published:** 2025-02-05

**Authors:** Marketa Husakova, Petra Patakova

**Affiliations:** † Department of Biotechnology, University of Chemistry and Technology Prague, Technicka 5, 160 00 Prague, Czech Republic

## Abstract

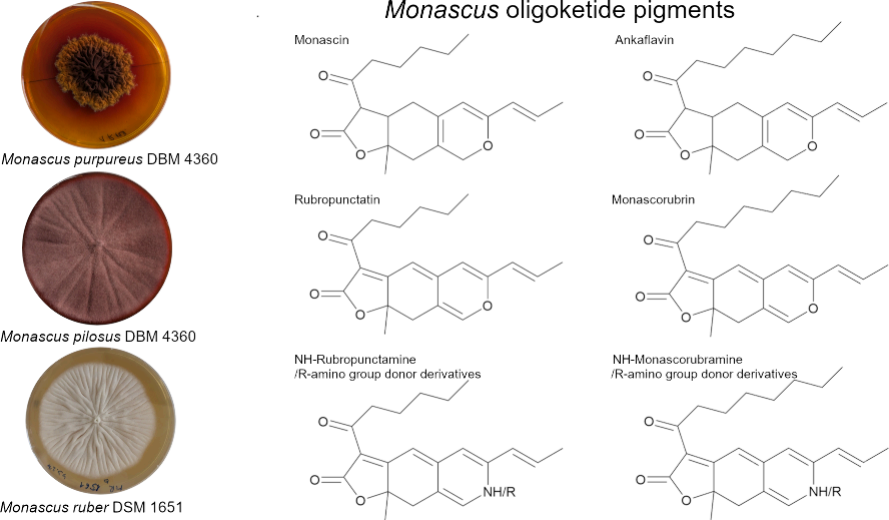

*Monascus* pigments having yellow, orange, and red colors are widely studied for their potential beneficial properties. Many different biological activities have been reported regarding *Monascus* pigments and their derivatives, but the usual method is to test complex extracts from the mycelium of the fungus or from a fungus-fermented substrate. However, this review is mainly concerned with the biological activities of purified *Monascus* pigments. Both yellow (ankaflavin, monascin) and red (rubropunctamine, monascorubramine) *Monascus* pigments are proven antioxidants if used in concentrations of 10 μg/mL or higher. Antimicrobial activity against Gram-positive and Gram-negative bacteria and fungi has been observed with all *Monascus* pigments. However, the best antimicrobials are red *Monascus* pigments, and their amino acid derivatives (l-cysteine derivatives have MIC 4 μg/mL against *Enterococcus faecalis*). Yellow monaphilones and orange monaphilols seem to have the highest anti-inflammatory activity (IC_50_ 1.7 μM of monaphilol D) and, together with red *Monascus* pigment derivatives, have mild antiobesity and antidiabetic activities. Further, monascin and ankaflavin in daily doses of 0.5 and 0.08 mg, respectively, lowered serum blood levels of low-density lipoprotein cholesterol complexes in rats on a high-fat diet. Orange *Monascus* pigments, rubropunctatin and monaphilols A and C, exhibit cytotoxic and antitumor activities (IC_50_ 8–10 μM).

## Introduction

*Monascus* pigments are compounds with an oligoketide structure, produced by representatives of the genus *Monascus*(1,2) and by some species of *Talaromyces/Penicillium*.^3^*Monascus* pigments are traditionally used for food dyeing and preservation,^1^ but as with many other natural products, they are also being studied for their potential health benefits. There is only one product on the world market, Ankascin 568,^4^ whose cholesterol lowering activity relates to yellow *Monascus* pigments, ankaflavin (**2**) and monascin (**1**), but not to monacolin K (known also as lovastatin, an uncolored statin compound that may be produced by specific *Monascus* strains). Unlike red yeast rice (RYR; rice fermented by the fungus *Monascus*) containing monacolin K (the same substance as in the prescribed medication), Ankascin 568 was approved by the FDA as a new dietary ingredient.

**Figure 1:**
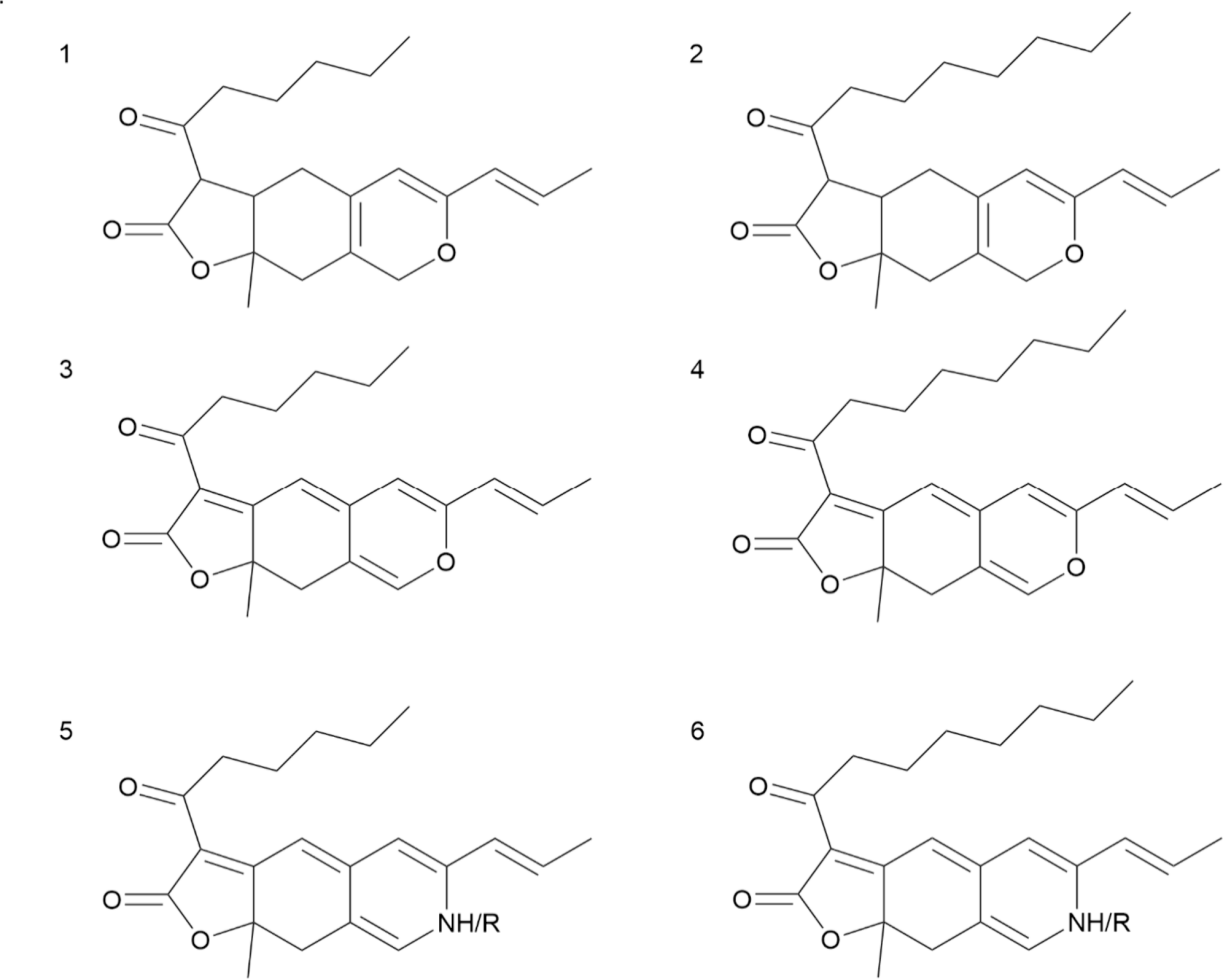
Typical *Monascus* pigments; yellow: monascin (**1**); ankaflavin (**2**); orange: rubropunctatin (**3**); monascorubrin (**4**); red: containing NH group – rubropunctamine (**5**); monascorubramine (**6**), or “R” other red derivatives containing an R-amino group donor.

**Figure 2:**
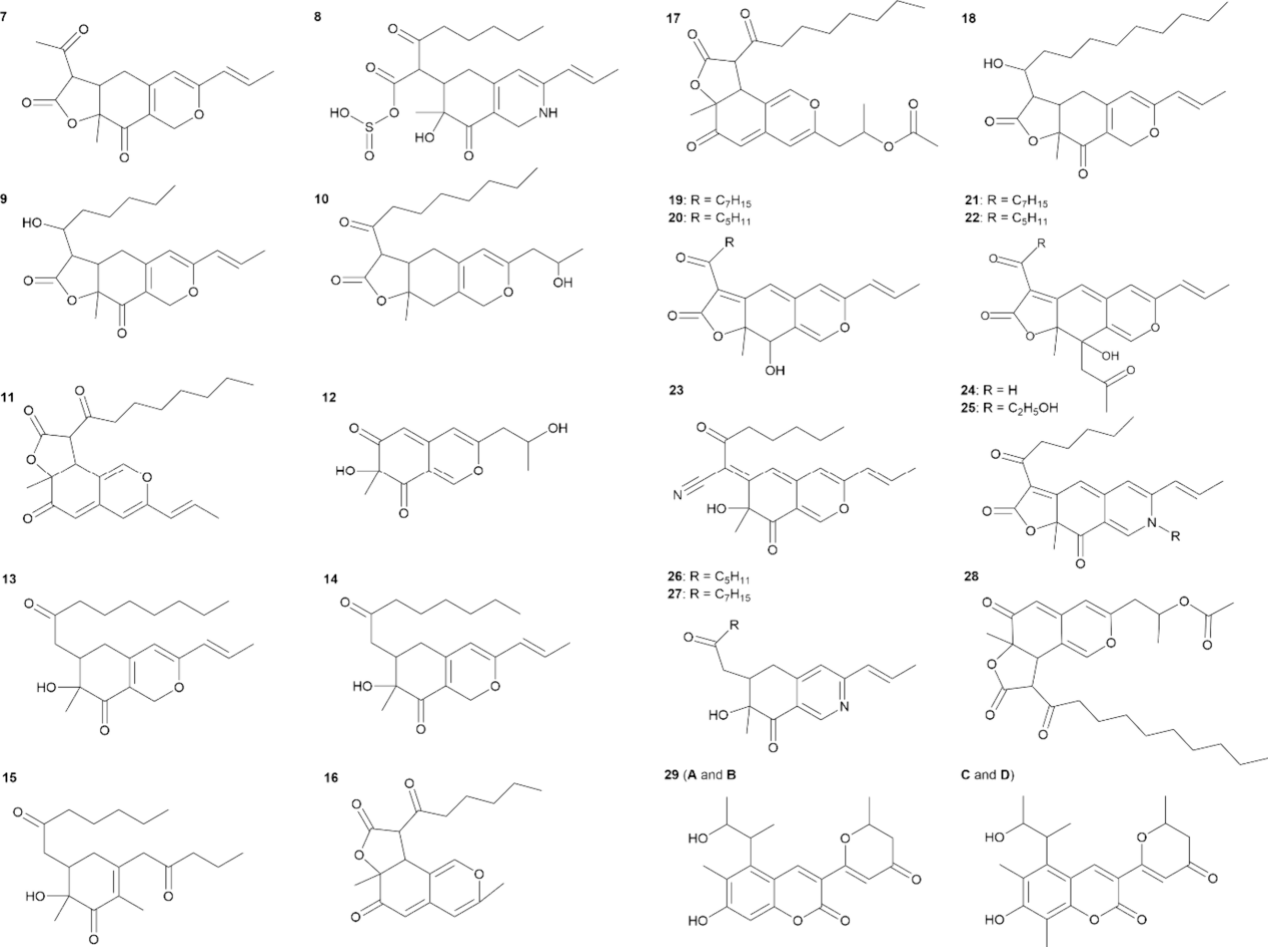
Atypical *Monascus* pigments mentioned in the text: monascusone B (**7**), C_21_H_27_NO_7_S (**8**), monascuspiloin (**9**), monasfluol B (**10**), monasfluore B (**11**), azanigerone E (**12**), monaphilones A–C (**13**, **14**, **15**), monascuskaolins A and B (**16**, **17**), monascusazaphilol (**18**), monaphilols A–D (**19**–**22**), monapilonitrile A (**23**), monapilosine (**24**), and *N*-ethanolic monapilosine (**25**), monascopyridines C and D (**26**, **27**), monascuskaolin (**28**), and monankarins A–D (**29**)*. *Monankarins A, B and C, D, respectively, are diastereomers.

Typical *Monascus* pigments, associated mainly with *Monascus purpureus*, include three subgroups differing in color: monascin (**1**) and ankaflavin (**2)** (yellow), rubropunctatin (**3**) and monascorubrin (**4**) (orange), rubropunctamine (**5**) and monascorubramine (**6**) (red), see Figure 1. Within this group, yellow and orange *Monascus* pigments are biosynthetically produced but the third subgroup–red *Monascus* pigments, are formed by the chemical reaction of orange *Monascus* pigments with compounds containing a free amino group. In this reaction, which is supposed to take place outside cells and to contribute to self-defense of the fungus against hostile challenges,^5^ the pyranyl oxygen of rubropunctatin (**3**) or monascorubrin (**4**) is replaced by a primary amine.^6,7^ This reaction with defined primary amine donors (such as polar/nonpolar or acidic/basic amino acids) may provide a pathway for producing *Monascus* pigment derivatives with specific properties.^8^ There have already been described more than 100 *Monascus* pigments^9^ while their formation may be associated not only with *M. purpureus* but also with other species of the genus *Monascus*, in particular *Monascus ruber* and *Monascus pilosus*.^10^ Their production may involve chemical modification of the typical *Monascus* pigments^5^ or can be based on mutation of *Monascus* strains.^11−13^ Atypical *Monascus* pigments mentioned in this study are shown in Figure 2.

The biological activity of *Monascus* pigments has been studied using crude extracts, containing both different *Monascus* pigments and other compounds, as well as using pure *Monascus* pigments and defined mixtures. However, the following bottlenecks must be addressed or at least considered during the testing of the biological activities of *Monascus* pigments:

- Since the separation and isolation of individual pigments are very complex processes,^14,15^ the biological activities of *Monascus* pigments are usually tested with crude extracts, which is problematic due to the unknown or only partially known composition of *Monascus* pigments, the synergistic action of multiple *Monascus* pigments and nonrepeatable results.
- The orange *Monascus* pigments, rubropunctatin (**3**) and monascorubrin (**4**), are highly reactive and may be converted to their red derivatives even during extraction, standardly performed using ethanol or methanol, if suitable amino group-containing reactants (e.g., amino acids) are coextracted. Therefore, extraction of pigments from mycelia or fermented material is sometimes done with acidified ethanol (pH 2 or 4) to prevent conversion to red pigments at low pH.^16^ As biological activity tests are frequently (but not always) performed under neutral pH and in an environment with free amino group-containing compounds or structures (peptides, proteins, cell surface etc.), a nonspecific conversion of orange to red *Monascus* pigments may result in their nonspecific biological effect (e.g., by binding to enzymes or cell surface).
- Among typical *Monascus* pigments, only red pigments are water-soluble, while yellow and orange pigments are not; they can, however, be dissolved in dimethyl sulfoxide,^17^ which is generally suitable for biological tests. Nevertheless, some studies mention atypical water-soluble yellow or orange *Monascus* pigments.
- The principles of biological activity tests are very frequently based on colorimetric reactions and *Monascus* pigments may interfere with the spectrophotometric analysis (at 300–600 nm) used for activity assessments. Often, therefore, assays based on other principles must be used, which makes rapid screening for biological activities particularly difficult.

Despite these problems, *Monascus* pigments seem to be promising natural biologically active compounds with low toxicity that may exhibit specific, repeatable and measurable effects. This review focuses mainly on pure *Monascus* pigments (see Figures 1 and 2). Since the typical orange *Monascus* pigments (**3** and **4**) activity was partially nonspecific or random (e.g., in antimicrobial or cytotoxicity tests), the description was moved to the Supporting Information file. The main areas of activities that are reviewed include antioxidant, antimicrobial, anti-inflammatory, antitumor, cytotoxic and antiobesity effects. Safety assessment of *Monascus* pigments^18−20^ was considered to be more related to their food applications, where the main issue is potential citrinin contamination, and, with the exception of cytotoxicity studies, is not covered in this review. Potential mechanisms of action of the different types of biological activities are also summarized in the individual sections of this review. Although several reviews^21−23^ have been published on the subject, we believe that a critical and focused evaluation of the biological activity of purified pigments provides a new perspective.

## Biological Activities of *Monascus* Pigments

### Antioxidant Activity

Antioxidants are compounds that are able to capture free radicals, prevent or inhibit oxidation, and protect sensitive macromolecules. Oxidative stress is one of the factors that causes or supports the deterioration of food or other natural products and the development of a large number of human diseases.^24,25^ Antioxidant activity may be tested *in vitro* and *in vivo*,^26^ focused on different antioxidant features and may therefore provide different results even for a single compound. Antioxidant effects of *Monascus* pigments may promote other biological effects; an example is the induction of apoptosis in gastric cancer cells by *Monascus* pigments, where the basis of this action is the scavenging of mitochondrial reactive oxygen species (ROS).^27^

The theoretical study performed by Thang et al.^28^ predicted the antioxidant capacities of the six main *Monascus* pigments (see Figure 1). Their antioxidant capacity was estimated to be in the following order: ankaflavin (**2**) > monascin (**1**) > rubropunctatin (**3**) > monascorubrin (**4**) > monascorubramine (**6**) > rubropunctamine (**5**).^28^ Koli et al.^29^ obtained contradictory results while testing the red pigment rubropunctamine (**5**) and a mixture of yellow pigments ankaflavin (**2**) and monascin (**1**). The antioxidant activity was 68% and 27% for 10 mg of rubropunctamine (**5**) and 3% and 15% for 10 mg of yellow pigments determined by the FRAP and the DPPH assays, respectively (the ascorbic acid standard represents 100%).^29^ Other published data show that 2.5 g/L of water-soluble yellow MPs (a mixture of monascusone B^7^ and a compound with the molecular formula C_21_H_27_NO_7_S (**8**)^30^) has an antioxidant capacity of 93% compared to 21% antioxidant capacity of a 110 μM alcoholic solution of yellow standards (monascin (**1**) and ankaflavin (**2**), 39 and 43 μg/mL, respectively).^31^ Other tests determined the antioxidant activity of 100 μg/mL of monascin (**1**) as 98% (the positive control of 7 μg/mL ascorbic acid had 38% antioxidant capacity) and it was determined that the antioxidant activity of *Monascus* pigments was proportional to their concentrations.^28^ The half maximal inhibitory concentration (IC_50_) determined for monascuspiloin (**9**) (yellow pigment isolated from *Monascus pilosus*)^32^ was 80 μg/mL, and for monasfluol B (**10**), 62 μg/mL;^33^ see the pigments structures in Figure 2. The antioxidant activity of complex *Monascus* extracts is referred to in the Supporting Information file.

Practical applications of *Monascus* pigments as antioxidants has already been tested in sunscreens,^29^ functional foods (rice noodles),^31^ functional alcoholic beverages^34^ or a variety of fermented plant substrates, typically rice but also others, such as adlay (*Coix lacryma-jobi*),^35^ waxy corn^36^ or green coffee beans^37^ meant for direct consumption. Antioxidant properties of *Monascus* pigments in fermented food are enhanced by antioxidants found in the substrate, e.g., phenolic acids and flavonoids in rice.^38^

### Antimicrobial Activity

Multidrug-resistant bacteria are a global problem for public health. According to the WHO, antimicrobial resistance (AMR) belongs to the top 10 global public health threats.^39^ One of the strategies to solve the AMR problem is to search for new antimicrobials and develop new methods of treatment.^40^

The antimicrobial activity of *Monascus* pigments against various microorganisms has been reported (see Table 1). However, the chosen type of antimicrobial test^41^ may affect the results. In general, Gram-positive bacteria are the most susceptible to *Monascus* pigments, regardless of the type, compared to Gram-negative bacteria, yeast and filamentous fungi.^42,43^ The most effective were red *Monascus* pigments and their derivatives, which were effective against Gram-positive and Gram-negative bacteria and also against fungi.^42,44−46^ The effectiveness of amino acid derivatives depends on the bound amino acid, with the hydrophobic amino acids exhibiting higher antimicrobial activities at lower doses than hydrophilic ones. The most effective were the red derivatives with the amino acid l-phenylalanine attached, which caused the formation of cell aggregates and blocked oxygen uptake, resulting in a limitation of bacterial growth. SEM and TEM images showed disruption of the bacterial cell surface.^44^ The potency of orange *Monascus* pigments was similar to that of red ones^46−49^ (see Supporting Information: Table S1) and the effect of yellow *Monascus* pigments (monascin **(1)**, ankaflavin (**2**)) against *Bacillus subtilis* was lower compared to red *Monascus* pigments.^47,50^ Antimicrobial activities of *Monascus* pigments, determined as the diameter of zones of inhibition, can be found in Supporting Information: Table S2. The antimicrobial effect of complex *Monascus* pigment extracts was influenced by fungal growth conditions; there was a high impact of cultivation type (submerged or solid), the type of substrate, the source of carbon and nitrogen^43,51^ and the selected *Monascus* strain (see Supporting Information: Table S1). Antifungal activity consisted of suppression of germination of *Aspergillus niger* conidia and interaction of pigments with the mycelial surface.^52^ The essence of the antimicrobial effect of *Monascus* pigments appears to be their interactions with the surface of microbial cells^49^ that can lead to potential disruption of membranes and blocking of transport phenomena^52^ including oxygen transfer into the cells.^44^

**Table 1:** Minimal Inhibition Concentration of Yellow and Red *Monascus* Pigments and Their Derivatives against Various Gram-Positive and Gram-Negative Bacteria and Fungi

		**amino acid derivatives of red*****Monascus*****pigment**							
**MIC [μg/mL]**	**red*****Monascus*****pigment**	l-Asp	d-Asp	l-Cys	l-Glu	l-Phe	d-Phe	l-Tyr	d-Tyr
**Gram-positive bacteria**									
*Bacillus subtilis* KCCM 11316^44^	>128	8	8	32		8	8	4	4
*Staphylococcus aureus* KCCM 11814^44^	64	8	16	16		8	8	8	8
*Staphylococcus aureus* KCCM 11817^44^	64	16	16	16		16	16	8	16
**Gram-negative bacteria**									
*Enterobacter aerogenes* KCCM 12177^44^	>128	16	16	8		16	16	4	16
*Enterococcus faecalis* KCCM 11814^44^	64	64	32	4		4	8	8	4
*Escherichia coli*(8)		128			64	16		8	
*Escherichia coli* KCCM 11234^44^	>128	16	16	64		8	8	16	16
*Haemophilus influenza* KCCM 11903^44^	64	32	32	8		16	16	32	16
*Proteus vulgaris* KCCM 11906^44^	>128	16	16	16		8	8	8	16
*Pseudomonas aeruginosa* KCCM 11328^44^	>128	8	8	16		8	16	8	16
*Salmonella choleraesuis* KCCM 11806^44^	64	8	8	16		8	4	8	4
*Salmonella typhimurium* KCCM 11806^44^	64	8	16	8		4	8	16	4
*Shigella sonnei* KCCM 40253^44^	>128	8	32	16		16	16	32	8
**fungi**									
*Aspergillus niger* KCCM 11239^44^	64	4	4	8		8	8	4	4
*Candida albicans* KCCM 10231^44^	32	4	8	16		8	8	8	16
*Penicillium citrinum* KCCM 11663^44^	32	8	8	32		8	8	8	4
*Penicillium digitatum* KCCM 60140^44^	32	32	32	32		16	16	32	32

### Special Antimicrobial Applications of *Monascus* Pigments

The red *Monascus* pigments were applied several times for coloring meat products,^53−55^ but there was one open question; can red *Monascus* pigments prevent the germination of *Clostridium* spores as well as nitrite salts traditionally used in meat products. The question was addressed by Husakova et al.^56^ and the inhibition of *Clostridium* spore germination by red yeast rice (RYR; rice fermented by the fungus *Monascus*) extracts in combination with NaCl was demonstrated. In addition, complex extracts of *Monascus* pigments were tested as photosensitizing agents efficient in antimicrobial photodynamic therapy (aPDT) against bacteria, and were confirmed as promising agents, especially against the growth of *E. coli* if the extracts contained monascuspiloin (**9**).^17^ The extracts with monascuspiloin (**9**) were ineffective in the dark but inhibited the growth of Gram-negative bacteria at a concentration of 4 μg/mL after irradiation with light of wavelength 410 nm. Also in this case, the main effect will probably be attributed to disruption of the cell surface.^17^ Further, red *Monascus* pigments were used to form and stabilize pigment-silver nanoparticles from silver nitrate under solar irradiation, which were confirmed as efficient inhibitors of *Pseudomonas aeruginosa*, *Staphylococcus aureus* and *E. coli*.^57^ Rubropunctatin (**3**)-functionalized silver nanoparticles^58^ were more efficient than standard silver nanoparticles against *E. coli* and *S. aureus* when they degraded bacterial cell membranes but exhibited lower cytotoxicity against mouse fibroblast cells compared to nonfunctionalized nanoparticles.

### Anti-Obesity and Anti-Diabetic Activity

Obesity and diabetes are noncommunicable diseases and, with their increasing prevalence, are a major public health problem.^59^ Furthermore, these diseases are risk factors for developing and promoting atherosclerosis, which can lead to the development of cardiovascular diseases.^60^ A promising treatment for dealing with obesity and diabetes is the use of various enzyme inhibitors.^60^ Specifically, inhibitors of pancreatic lipases, α-amylases and α-glucosidases such as acarbose are commercially used in the prevention and treatment of diabetes.^61^ Lipases (triacylglycerol acyl hydrolase, EC 3.1.1.3) are digestive enzymes involved in fat absorption by hydrolyzing triglycerides into diglycerides, monoglycerides, and free fatty acids.^62^ α-Amylases (EC 3.2.1.1) are endoenzymes involved in starch digestion, i.e., the cleavage of α-1,4-glycosidic bonds in amylose or amylopectin.^63^ α-Glucosidases (α-d-glucoside glucohydrolase, EC 3.2.1.20) are exoenzymes that catalyze the release of d-glucose from the nonreducing end of the substrate.^64^ Yellow pigments (monascin (**1**) and ankaflavin (**2**)) and monasfluore B (**11**) (Figure 2) were identified as noncompetitive inhibitors of pancreatic lipases^65^ and red *Monascus* pigment derivatives with aromatic and nonpolar aliphatic amino acids showed low IC_50_ values; for l-Trp and d-Tyr derivatives of 61 and 103 μM, respectively. The most effective inhibitor with specific activity against pancreatic lipase was a derivative with a bound modified amino acid (l-leucine ethyl ester).^66^ Red derivatives with chemically synthesized amino acids are noncompetitive inhibitors of lipase; the most effective being derivatives with butylglycine (IC_50_ 170 μM), cyclohexylalanine (IC_50_ 42 μM), and penicillamine (IC_50_ 24 μM).^67^ Red derivatives with the chemically synthesized amino acid penicillamine are mixed type inhibitors of α-glucosidase with an IC_50_ value of 50 μM.^67^

In addition to enzyme inhibition, the mode of action of the lipid lowering effect can be connected to antagonism of peroxisome proliferator activated receptors (PPARs). Monascin (**1**) and ankaflavin (**2**) are possible agonists of PPAR-α and PPAR-γ.^68−73^ PPARs (PPAR-α, PPAR-β PPAR-γ) are transcription factors that control genes involved in adipogenesis, lipid metabolism (fatty acid oxidation), inflammation, and maintenance of metabolic homeostasis. Especially PPAR-α and PPAR-γ are molecular targets for lipid-lowering drugs and for insulin-sensitizing thiazolidinedione (antidiabetic agent for type 2 (noninsulin dependent) diabetes mellitus treatment).^74,75^

Another potential target for type 2 diabetes treatment is the protein tyrosine phosphatase 1B (PTP1B), which is a regulator of the insulin and leptin signaling pathways,^76^ or cholesteryl ester transfer protein (CETP), which facilitates the transfer of cholesteryl esters and triglycerides between lipoproteins (from HDL to LDL).^77^ The crude extract of red yeast rice (RYR; rice fermented by the fungus *Monascus*) was determined as a PTP1B inhibitor with an IC_50_ of 7.56 μg/mL, and monascorubramine (**6**) was identified as the active compound according to binding specificity.^78^ Red amino acid (l-Thr and l-Tyr) *Monascus* pigment derivatives exhibited CETP inhibition with IC_50_ values of 1.0 and 2.3 mM respectively.^79^

Obesity and diabetes are often linked to cardiovascular disease. In addition to monacolins, the known inhibitors of HMG-CoA reductase (a key enzyme for *de novo* synthesis of cholesterol) produced by *Monascus* fungi, *Monascus* pigments and *Monascus* fermented products in general are considered to be possible antiobesity and antidiabetic agents.^80^ The blood serum of high-fat diet-fed rats, to which were administered 0.55 mg of monascin (**1**) or 0.08 mg of ankaflavin (**2**) daily for 8 weeks, exhibited a decrease in the low-density lipoprotein cholesterol (LDL-C) to a high-density lipoprotein cholesterol (HDL-C) ratio of about 40 and 54% compared to monacolin K administered in a daily dose of 0.16 mg.^81^ Daily doses for rats were designed to mimic human intake of monascin (**1**) and ankaflavin (**2**) in one Ankascin tablet per day while the study demonstrated that monascin (**1**) and ankaflavin (**2**) suppressed assembly of LDL-C and stimulated the expression of apo A1 lipoprotein.^81^ The observation was also confirmed in a clinical trial with Ankascin 568.^82^ The similar effect of ankaflavin (**2**) or monascin (**1**) administration resulting in a reduction of total cholesterol and a preventive increase of HDL-C were observed.^68,69^

Overall, all types of *Monascus* pigments (yellow (**1, 2**), orange (**3, 4**), and red **(5**, **6**)) were confirmed to be potential agents to prevent or treat metabolic disorders, obesity and diabetes in a study where rats on a high-fat diet were fed with 20 mg of each pigment in a daily dose.^83^ All pigments (**1**–**6**) lowered blood serum LDL-C but the yellow pigments (**1, 2**) had the most pronounced effect, cf. 36% drop for (**1, 2**) and 15% drop for (**3**–**6**) in comparison with the control. In addition, all pigments (**1**–**6**) reduced the blood glucose concentration by 15% compared to the control.

### Cytotoxicity and Antitumor Activity

Cancer comprises a number of noncommunicable diseases that cause millions of deaths every year and affect almost every organ or tissue.^84^ Despite ongoing research, the continuing need for new anticancer drugs or alternative treatment modalities persists. Therefore, many natural products, including *Monascus* pigments, are being tested as possible antitumor and anticancer agents.

Conventional *Monascus* pigments and their derivatives have been tested as antiproliferative agents against different types of normal and cancer cell lines, and *in vivo* tests on mice models were also performed.

Both yellow and orange *Monascus* pigments demonstrate inhibitory activities in *in vitro* assays on various cancer cell lines with IC_50_ values between 10 and 100s of μM. The antiproliferative activity evaluated as half maximal inhibitory concentrations (IC_50_), is shown in Table 2. Furthermore, yellow *Monascus* pigments (monascin (**1**),^85^ monascuspiloin (**9**),^32^ and water-soluble yellow pigments (a mixture of azanigerone E (**12**) and three unidentified compounds with molecular weights of 254, 402, and 358 g/mol)^86,87^) and complex RYR extracts^88,89^ caused growth suppression and various levels of growth inhibition (21–60%); toxicity was not observed in control cell lines (RAW 264.7 cells,^88,90^ MRC- cells,^91^ murine NMuMG normal breast cells,^85^ normal human lung cell lines WI-38 and MRC-5.^11,91^) Compared to the standard chemotherapeutic agent mitomycin C, cytotoxicity of yellow *Monascus* pigments (ankaflavin (**2**) and monascin (**1**)) and yellow derivatives (monaphilones A-C (**13**, **14**, **15**)) was poor.^11^ However, rubropunctatin (**3**), in combination with irradiation, was more effective than Taxol against HeLa cells^92^ and seemed to be a promising dual agent for chemotherapy and phototherapy that induced apoptosis in treated cells. *In vivo* assays were performed with yellow *Monascus* pigments (monascin (**1**)^93^ and monascuspiloin (**9**)^32^), orange *Monascus* pigment (monascorubrin (**4**)^94^) and with a complex RYR extract.^95^ Antitumor activity was tested against skin and prostate tumors; all *Monascus* pigments tested suppressed tumor formation and growth, and no toxicity was observed in treated animals.^32,93−95^ The cytotoxic and antitumor mechanisms of action of *Monascus* pigments included cell cycle arrest, apoptosis induction, inhibition of proliferation or angiogenesis and induction of autophagy.^96^

**Table 2:** Half-Maximal Inhibitory Concentrations of Yellow and Orange *Monascus* Pigments Effective against Proliferation of Various Cancer Cell Lines

***Monascus*****pigment**	**color**	**cell line**	**IC**_**50**_**[μM]**
ankaflavin (**2**)^91^	Y	A549 human cancer cell lines	38.81
ankaflavin (**2**)^91^	Y	Hep G2 human cancer cell lines	38.81
ankaflavin (**2**)^11,100^	Y	HEp-2 (human laryngeal carcinoma cell line)	31.62–94.7
ankaflavin (**2**)^11^	Y	WiDr (human colon adenocarcinoma cell line)	111.6
monaphilone A (**13**)^11,100^	Y	HEp-2 (human laryngeal carcinoma cell line)	20.97–72.1
monaphilone A (**13**)^11^	Y	WiDr (human colon adenocarcinoma cell line)	55.8
monaphilone B (**14**)^11^	Y	HEp-2 (human laryngeal carcinoma cell line)	77.6
monaphilone B (**14**)^11^	Y	WiDr (human colon adenocarcinoma cell line),	55.3
monaphilone C (**15**)^11^	Y	HEp-2 (human laryngeal carcinoma cell line)	124.1
monaphilone C (**15**)^11^	Y	WiDr (human colon adenocarcinoma cell line)	142.4
monaphilol A (**19**)^90^	O	HEp-2 (human laryngeal carcinoma cell line)	9.8
monaphilol A (**19**)^90^	O	WiDr (human colon adenocarcinoma cell line)	8.6
monaphilol B (**20**)^90^	O	HEp-2 (human laryngeal carcinoma cell line)	14.8
monaphilol B (**20**)^90^	O	WiDr (human colon adenocarcinoma cell line)	15.7
monaphilol C (**21**)^90^	O	HEp-2 (human laryngeal carcinoma cell line)	8.7
monaphilol C (**21**)^90^	O	WiDr (human colon adenocarcinoma cell line)	11
monaphilol D (**22**)^90^	O	HEp-2 (human laryngeal carcinoma cell line)	10.4
monaphilol D (**22**)^90^	O	WiDr (human colon adenocarcinoma cell line)	10.5
monascin (**1**)^11^	Y	HEp-2 (human laryngeal carcinoma cell line)	59.8
monascorubrin (**4**)^90^	O	HEp-2 (human laryngeal carcinoma cell line)	55.9
monascorubrin (**4**)^90^	O	WiDr (human colon adenocarcinoma cell line),	54.1
monascuspiloin (**9**)^101^	Y	LNCaP androgen-dependent human prostate cancer cells	44.97
monascuspiloin (**9**)^101^	Y	PC-3 androgen-independent human prostate cancer cells	46.96
rubropunctatin (**3**)^90^	O	HEp-2 (human laryngeal carcinoma cell line)	106.8
rubropunctatin (**3**)^90^	O	WiDr (human colon adenocarcinoma cell line)	109.3
rubropunctatin (**3**)^102^	O	AGS human gastric adenocarcinoma cells	7.98
rubropunctatin (**3**)^102^	O	BGC-823 human gastric adenocarcinoma cells	12.57
rubropunctatin (**3**)^92^	O	HeLa cell line	93.71
rubropunctatin (**3**) in combination with irradiation^92^	O	HeLa cell line	24.02
rubropunctatin (**3**)^102^	O	HepG2 hepatocellular carcinoma cell	44.19
rubropunctatin (**3**)^102^	O	HT-29 colon cancer cell	36.69
rubropunctatin (**3**)^102^	O	MKN45 human gastric adenocarcinoma cells	14.27
rubropunctatin (**3**)^102^	O	SH-SY5 neuroblastoma cell	30.95

The limitations of possible clinical applications are poor water solubility and stability. Therefore, liposomes^97,98^ and nanoparticles^99^ have been proposed as *Monascus* pigment carriers. The incorporation of *Monascus* pigments into liposome carriers or the loading of *Monascus* pigments into nanoparticles enhanced their stability, water solubility, cytotoxicity and antitumor activity.^97−99^

### Anti-Inflammatory Activity

Inflammation has been characterized as the body’s reaction to tissue damage and infection. However, increased levels of inflammatory molecules and cells can be observed under various conditions without tissue damage or infection. Therefore, inflammation was redefined as the innate immune response to potential threats such as pathogens, injury, and metabolic stress, rather than simply the response to tissue injury or infection. The inflammatory pathway includes a variety of signaling molecules, transcription factors, cytokines, and enzymes.^103^

*Monascus* pigments have been considered anti-inflammatory agents. The main activity is inhibition of nitric oxide production (see Table 3); nitric oxide is formed by endothelial cells as a signaling molecule. In abnormal situations, overproduction of nitric oxide causes inflammation by inducing vasodilation and immune responses.^104^ Other signaling molecules involved in immune responses are cytokines, and the levels of cytokines have been measured (e.g., tumor necrosis factor-α, interleukin-1β, and interleukin-6). Cytokine levels were significantly reduced by yellow *Monascus* pigments (monascin (**1**), ankaflavin (**2**),^105,106^ monascuskaolin A (**16**), monascuskaolin B (**17**), monasfluol B (**10**),^107^ monascusazaphilol (**18**)^108^), orange *Monascus* pigments (monascorubrin (**4**), rubropunctatin (**3**), monaphilol A (**19**), monaphilol B (**20**), monaphilol C (**21**), monaphilol D (**22)**^105^) (Figure 2), and complex extracts.^109−111^ Yellow *Monascus* pigments (monascin (**1**), ankaflavin (**2**)) also affected transcription factors NF-κB involved in the immune response.^106^

**Table 3:** Half-Maximal Inhibitory Concentrations of Yellow and Red *Monascus* Pigments and Their Derivatives Effective against Nitric Oxide Production Stimulated by Lipopolysaccharides in RAW 264.7 Cells

***Monascus*****pigment**	**color**	**IC**_**50**_**[μM]**
ankaflavin (**2**)^100,105,112^	Y	21.73–67.89
monaphilone A (**13**)^105^	Y	19.69
monaphilone B (**14**)^105^	Y	22.56
monapilonitrile A (**23**)^112^	R	2.60
monapilosine (**24**)^112^	R	12.51
monaphilol A (**19**)^90,105^	O	0.99–1.04
monaphilol B (**20**)^90,105^	O	3.65–3.79
monaphilol C (**21**)^90,105^	O	2.72–2.79
monaphilol D (**22**)^90,105^	O	1.7
monascin (**1**)^100,105,112^	Y	19.70–48.10
monascopyridine C (**26**)^113^	R	57.05
monascopyridine D (**27**)^113^	R	74.74
monascorubrin (**4**)^105^	O	>39.23
monascuskaolin (**28**)^113^	Y	16.62
*N*-ethanolic monapilosine (**25**)^112^	R	27.49
rubropunctatin (**3**)^105^	O	21.16

### Other Biological Activities

In addition to the above-described activities, *Monascus* pigments may be used as alternative antidepressants. Monankarins (**29**), the derivatives of *Monascus* pigments with a pyrano-coumarin skeleton, inhibit monoamine oxidase, in particular monankarin C and A have IC_50_ values 10.7 and 15.5 μM, respectively.^12^ Monoamine oxidases catalyze the oxidative degradation of biogenic amines and neurotransmitters, such as norepinephrine, serotonin, dopamine or tyramine.^114^

Yellow *Monascus* pigments (ankaflavin (**2**) and monascin (**1**)) and *Monascus* fermented products were tested on rats and described as suppressors of risk factors for Alzheimer’s disease, namely lowering accumulation of amyloid β peptide in brain, and could improve memory and learning ability.^115−118^

Some *Monascus* pigments have also been reported as potential photoprotective agents^29,118^ and derivatives of synthetic orange *Monascus* pigments as inhibitors of melanogenesis.^119^

## Conclusion

The *Monascus* fungi have been known and used in Chinese traditional medicine for centuries. At first glance, there seems to be an overwhelming number of articles dealing with the biological effects of *Monascus* metabolites, especially pigments. Unfortunately, on closer examination, it turns out that most of the articles deal with the biological activity of complex extracts from the mycelium of various fungal species or from the substrate fermented by these fungi. With few exceptions, these communications were excluded from this review due to the inability to compare their results with other studies. However, this approach may be risky, as it should be taken into account that complex extracts from *Monascus* or from substrates fermented with this fungus might be more effective than pure substances, due to the synergistic effect of two or more substances in these extracts.

The study of pure pigments isolated from different fungi of the genus *Monascus* is only at the beginning and individual studies are difficult to compare because their authors have chosen different concentrations, application conditions, or methods of evaluating the result. Also, not all studies correctly document the statistical significance of the observed findings. However, the main thing that hinders the further development of the field is the difficult, typically multistep isolation and purification of pure pigments, as well as the nonstandard culture conditions under which the fungus forms various metabolites. In this relation, optimizing cultivation and purification processes is essential for efficient screening of biological activities. Especially in-depth studies on the mechanisms of action of individual *Monascus* pigments are needed to better understand their functions and to evaluate their full potential.

Clinical trials in the field mainly compared the administration of statins–drugs, with a dietary supplement – *Monascus* red yeast rice, which contains monacolins and pigments. The usual conclusion is the demonstration of a certain but lower effect of the dietary supplement with fewer side effects on the patients with hypercholesterolemia compared to statin therapy. In other indications, *Monascus* pigments have probably not yet been tested on humans, but only using animal models, cell lines or microorganisms.

The review shows that pigments are promising antioxidants that can be applied together with their coloring effect in foods and dietary supplements. In addition, their antimicrobial activity, particularly evident in red pigments, may also be valuable for use in foods as well as their mild antiobesity and antidiabetic effects. The anti-inflammatory activity of the yellow and orange monaphilols (**19**-**22**) is of particular importance for the development of new drugs, as is the positive effect of yellow pigments, monascin (**1**) and ankaflavin (**2**) on the spectrum of serum lipid complexes with cholesterol. Other biological activities, such as cytotoxicity and antitumor activity, require further research, while the combined application of pigments with irradiation and the use of the photodynamic effect could be of particular interest.

## Supporting Information Available

The biological activities of orange *Monascus* pigments and complex *Monascus* extracts (PDF)
