# Supplementary material for: Purified Monascus Pigments: Biological Activities and Mechanisms of Action
Source: J Nat Prod. 2025 Feb 5;88(2):607–15. doi: 10.1021/acs.jnatprod.4c01008 (PMC11877510; doi:10.1021/acs.jnatprod.4c01008)
Supplement: Supplementary file 1 — np4c01008_si_001.pdf [file np4c01008_si_001.pdf]

Supporting Information

Purified *Monascus* Pigments: Biological Activities and Mechanisms of Action

Marketa Husakova[1], Petra Patakova[1*]

[1] Department of Biotechnology, University of Chemistry and Technology Prague, Technicka 5, 160 00 Prague, Czech Republic

[*]Corresponding author: petra.patakova@vscht.cz

Subtitle: The biological activities of orange *Monascus* pigments and complex *Monascus* extracts

Table of Contents:

Antioxidant Activities of *Monascus* Complex Extracts 1
Antimicrobial Activity 2
Table S1. Antimicrobial Activity of Orange *Monascus* Pigments and Complex *Monascus* Extracts 2
Table S2. Inhibition Zones Caused by Different *Monascus* Pigments and Complex *Monascus* Extracts Against Various Gram-positive, Gram-negative Bacteria, and Fungi 4
References 7

Antioxidant Activities of *Monascus* Complex Extracts

The antioxidant capacity of extracts from different *Monascus* species was determined and some differences were observed. *M. anka*, *M. ruber* and *M. purpureus* scavenged more than 60% of DPPH radicals and *M. pilosus* only about 20 to 39%. (1) The extracts from coloring and functional Qu (rice fermented with *Monascus*) showed an IC_{50} of 176 and 544 mg/L for coloring and functional Qu, respectively. The IC_{50} of ascorbic acid was 23 mg/L. The difference is defined by the presence of a larger amount of *Monascus* pigments in coloring Qu compared to the functional one, which is rich in monacolins. (2) Further, a complex methanolic extract containing yellow (monascin (**1**)), orange (rubropunctatin (**3**)) and red (rubropunctamine (**5**)) *Monascus* pigments, red derivatives and unusual *Monascus* pigments (e.g. monaphilone C(**15**), monasfluore B(**11**)) showed an IC_{50} 14.40 mg/L. (3)

Antimicrobial Activity

Table S1. Antimicrobial Activity of Orange *Monascus* Pigments and Complex *Monascus* Extracts

	MIC [mg/L]					
		complex extracts				
	**orange *Monascus* pigments**	RYR (hexan)	RYR (ethyl acetate)	RYR (ethanol)	mycelium (water)	RYR (ethyl acetate)
**gram-positive bacteria**						
Bacillus subtilis (4)	10					
Bacillus cereus ATCC 11778 (5)					128000	
Corynebacerium sp. 3B (4)	5					
Staphylococcus aureus ATCC 25923 (5)					256000	
Staphylococcus epidermidis ATCC 12228(5)					128000	
Streptomyces(4)	10					
**gram-negative bacteria**						
Agrobacterium MI (4)	20					
Enterococcus faecalis ATCC 29212 (5)					128000	
Enterococcus faecium DSM 13590 (5)					128000	
Escherichia coli ATCC 8739 (6)	2500					
Escherichia coli O157:H7 ATCC 35150 (5)					>256000	
Listeria *monocytogenese* Scott A (5)					256000	
Listeria innocua ATCC 33090 (5)					256000	
Propionibacterium acnes ATCC 11827 (7)						12500
Pseudomonas CCM 2115 (4)	7					
Pseudomonas CCM 2798 (4)	70					
Pseudomonas CCM 1640 (4)	<100					
Pseudomonas aeruginosa ATCC 27853 (5)					>25600	
Salmonella typhimurium CCM 5445 (5)					256000	
Salmonella typhimurium NRRL B-4420 (5)					256000	
**fungi**						
Aspergillus niger (4)	>100					
Aspergillus niger (food isolate) (5)					>259000	
Aspergillus niger MTCC 8652 (8)		1563.3	780	1563.3		
Aspergillus tereus (4)	>100					
Candida albicans ATCC 10239 (5)					>257000	
Candida pseudotropicalis (4)	30					
Candida tropicalis RSKK 2412 (5)					>256000	
Mucor rammonianus (4)	>100					
Myrothecium verrucaria (4)	30					
Saccharomyces cerevisisae (4)	30					

Saccharomyces cerevisiae (food isolate) (5)					>258000
Penicillium expansum MTTC 4900 (8)		1563.3	780	1563.3	
Penicillium nigricans (food isolate) (5)					>260000
Penicillium notatum (4)	>100				
Rhizopus stolinefer MTCC 10595 (8)		1563.3	780	1563.3	

Table S2. Inhibition Zones Caused by Different *Monascus* Pigments and Complex *Monascus* Extracts Against Various Gram-positive, Gram-negative Bacteria, and Fungi

	inhibition zones [mm]													
	**yellow *Monascus* pigments**			**orange *Monascus* pigments**				**red *Monascus* pigments**	**complex extracts**					
	monascin (**1**) 3000 mg/L	ankaflavin (**2**) 4000 mg/L	100µL	rubropunctatin (**3**) 1000 mg/L	monascorubrin (**4**) 2000 mg/L	10000 mg/L	100 µL	110000 mg/L	mycelia	mycelia	mycelia	culture media	RYR (ethyl acetate)	RYR (ethanol)	culture media
**gram-positive bacteria**														
Bacillus sp. (9)			14												16
Bacillus subtilis (10)												9			
Bacillus subtilis (11)												5.29			
Bacillus subtilis (12)												20			
Bacillus subtilis (13)	>10	>10		>10	>10										
Bacillus subtilis MCM B-310 (14)								1.36							
Bacillus megaterium MCM B-357 (14)								1.26							
Bacillus mycoides MCM B-358 (14)								1.34							
Staphylococcus albus (11)												1.66			
Staphylococcus aureus (12)												19			
Staphylococcus aureus (9)			18												

Staphylococcus aureus ATCC 6538 (15)		34								
Staphylococcus aureus MTCC 737 (3)				5.77	9.33	5.45				
MRSA ATCC BAA 811 (3)				6.7	6.42	3				
MRSA ATCC BAA-1683 (7)								10.48	9.75	
Streptomyces albus (10)							8			
**gram-negative bacteria**										
Escherichia coli ATCC 8739 (6)		21.3								
Escherichia coli (9)	12									
Escherichia coli (9)										18
Escherichia coli (11)							2.14			
Escherichia coli (12)							13			
Klebsiela sp. (9)	14									
Klebsiela sp. (9)										17
Propionibacterium acnes ATCC 11827 (7)								10.35	9.53	
Proteus sp. (9)	13	12								
Proteus sp. (9)										19
Providencia sp. (9)	14									
Pseudomonas sp. (9)	12									
Pseudomonas sp. (9)										24
Pseudomonas aeruginosa (10)							9			

Salmonella typhimurium MTCC 734 (3)				5.62 7.68 4.33
Vibrio cholerae N 16961 (3)				6.14 6.9 2.78
**fungi**				
Candida pseudotrpocialis (13)		>10 >10		

References

1. Aniya, Y.; Yokomakura, T.; Yonamine, M.; Shimada, K.; Nagamine, T.; Shimabukuro, M.; et al. Screening of antioxidant action of various molds and protection of *Monascus anka* against experimentally induced liver injuries of rats. *General Pharmacology: The Vascular System* **1999**, *32*(2), 225–231. doi:10.1016/S0306-3623(98)00183-9.
2. Wu, L.; Zhou, K.; Chen, F.; Chen, G.; Yu, Y.; Lv, X.; et al. Comparative study on the antioxidant activity of *Monascus* yellow pigments from two different types of hongqu—functional qu and coloring qu. *Frontiers in Microbiology* **2021**, *12*(2127). doi:10.3389/fmicb.2021.715295.
3. Kaur, M.; Goel, M.; Mishra, R. C.; Lahane, V.; Yadav, A. K.; Barrow, C. J. Characterization of the red biochromes produced by the endophytic fungus *Monascus purpureus* CPEF02 with antimicrobial and antioxidant activities. *Fermentation* **2023**, *9*(4), 328. doi:10.3390/fermentation9040328.
4. Martinkova, L.; Juzlova, P.; Vesely, D. Biological activity of polyketide pigments produced by the fungus *Monascus*. *Journal of Applied Bacteriology* **1995**, *79*(6), 609–616. doi:10.1111/j.1365-2672.1995.tb00944.x.
5. Gökmen, G. G.; Şılbır, M. S.; Göksungur, Y.; Kışla, D. Antimicrobial activity of red pigments derived from *Monascus purpureus*: A comparison to industrial red pigments. *JSFA reports* **2021**, *1*(1), 5–10. doi:10.1002/jsf2.20.
6. Guo-Ping, Z.; Ying-Qiu, L.; Jie, Y.; Kai-Yu, C. Antibacterial characteristics of orange pigment extracted from *Monascus* pigments against *Escherichia coli*. *Czech Journal of Food Sciences* **2016**, *34 (2016)*(No. 3), 197–203. doi:10.17221/430/2015-CJFS.
7. Milanda, T.; Zuhrotun, A.; Nabila, U.; Gathera, V. A.; Kusuma, A. S. Antibacterial activity of red yeast rice extract against *Propionibacterium acnes* ATCC 11827 and methicilin-resistant *Staphylococcus aureus* ATCC BAA-1683. *Pharmacology and Clinical Pharmacy Research* **2021**, *6*(2), 83–93.
8. Majhi, S.; Dhale, M. A.; Honganoor Puttananjaiah, M. Inhibitory effect of *Monascus purpureus* pigment extracts against fungi and mechanism of action. *Frontiers in Sustainable Food Systems* **2023**, *7*. doi:10.3389/fsufs.2023.1100961.
9. Gajalakshmi, P. Efficacy of polyketide pigment produced by *Monascus purpureus* and its biological activity. *International Journal on Nutraceuticals, Functional Foods and Novel Foods* **2019**. doi:https://doi.org/10.17470/NF-019-0016.
10. Ungureanu, C.; Ferdes, M. Antibacterial and antifungal activity of red rice obtained from *Monascus purpureus*. *Chemical Engineering Transactions* **2010**, *20*, 223–228.
11. Xu, W. Study on the liquid fermentation to produce *Monascus* pigment with corn starch and antibacteria. *Advanced Materials Research* **2011**, *183*, 1336–1340. doi:10.4028/www.scientific.net/AMR.183-185.1336.
12. Gao, X.; Lu, X.; Wang, Z.; Liu, G.; Li, X. Study on the extraction and antibacterial activity of Monascin. *E3S Web of Conferences* **2021**, *251*, 02061. doi:10.1051/e3sconf/202125102061.
13. Martinkova, L.; Patakova-Juzlova, P.; Kren, V.; Kucerova, Z.; Havlicek, V.; Olsovsky, P.; et al. Biological activities of oligoketide pigments of *Monascus purpureus*. *Food Additives & Contaminants* **1999**, *16*(1), 15–24. doi:10.1080/026520399284280.
14. Mukherjee, G.; Singh, S. K. Purification and characterization of a new red pigment from *Monascus purpureus* in submerged fermentation. *Process Biochemistry* **2011**, *46*(1), 188–192. doi:10.1016/j.procbio.2010.08.006.

15. Feng, L. H.; Li, Y. Q.; Sun, G. J.; Zhao, X. Z. Antibacterial effect of orange *Monascus* pigment against *Staphylococcus aureus*. *Acta Alimentaria* **2019**, *48*(2), 169–176. doi:10.1556/066.2019.48.2.4.
